# Dataset of full-length transcriptome assembly and annotation of a two-year-old *Bupleurum chinense* DC

**DOI:** 10.1016/j.dib.2026.113347

**Published:** 2026-10-13

**Authors:** Yanxia Gao, Binbin Wang, Jing Wang, Yan Xie, Jiahui Wang, Lan Yang, Gaixia Zhang, Hui Wang

**Affiliations:** a Sericulture Research Institute, Chengde Medical University, Chengde, 067000, China; b School of Basic Medical Sciences, Chengde Medical University, Chengde, 067000, China; c Department of Traditional Chinense Medicine, Chengde Medical University, Chengde, 067000, China; d College of Pharmacy, Shandong University of Traditional Chinense Medicine, Jinan, 250355, China

**Keywords:** *Bupleurum Chinense DC*, Full-length transcriptome, Gene functional annotation, SSR

## Abstract

Transcriptome sequencing has become an important tool for gene discovery in medicinal plants. Compared with next-generation sequencing (NGS), third-generation sequencing, also known as single-molecule real-time (SMRT) sequencing, provides longer reads and avoids PCR amplification, making it particularly suitable for full-length transcriptome analysis. *Bupleurum chinense* DC. is an important medicinal herb in China. However, genome information for *B. chinense* remains limited. In this study, total RNA was separately extracted from the root, stem, and leaf of a two-year-old *B. chinense* plant, and equal amounts of RNA from the three tissues were pooled for PacBio SMRT full-length transcriptome sequencing. This study generated the first full-length transcriptome dataset for *B. chinense*. The dataset includes 11,448 annotated unigenes, 690 transcription factors, and 6178 SSR markers, with many unigenes associated with terpenoid biosynthesis and other secondary metabolism pathways. The raw data were deposited in the SRA database under BioProject accession number PRJNA1494021. This dataset will facilitate gene discovery, functional characterization, saikosaponin biosynthesis research, marker development, and molecular breeding in *B. chinense* and related species.

Specifications Table

**Table:** 

Subject	Biology
Specific subject area	Medicinal Plant Transcriptome Analysis
Type of data	Table, Figure, Raw, Processed
Data collection	The samples of root, stem and leaf of *B. chinense* were processed using E.Z.N.A.® HP Total RNA Kit (Omega Bio-tek, USA). Only RNA samples with RIN ≥ 7.0 were retained. RNA purity and concentration were determined by a Nanodrop 2000 spectrophotometer (Thermo Fisher Scientific, USA) and a Qubit 3 Fluorometer (Thermo Fisher Scientific, USA), respectively. RNA was sequenced on a PacBio Sequel II platform using Single Molecule Real-Time(SMRT)technology. Raw PacBio sequencing data were processed using SMRT Link (v11.0) and the Iso-Seq3 pipeline to obtain full-length transcript sequences*.*
Data source location	Root, stem and leaf of *B. chinense* DC. were collected at: • Chengde Medical University • City/Town/Region: Anyuan Road, Shuangqiao District, Chengde • Country: China
Data accessibility	Repository name: National Center for Biotechnology Information (NCBI) Data identification number: https://www.ncbi.nlm.nih.gov/ under the accession number PRJNA1494021 Direct URL to data: https://www.ncbi.nlm.nih.gov/bioproject/PRJNA1494021 The BioSample and Sequence Read Archive (SRA) numbers are SAMN61584846 and SRR39583740, respectively.
Related research article	‘none’*.*

## 1 Value of the Data

• This dataset provides the first full-length transcriptome assembly of cultivated *Bupleurum chinense* generated using single molecule real-time (SMRT) sequencing.
• This dataset facilitates the identification of genes involved in the biosynthesis of key medicinal components including saikosaponins, volatile oils, polysaccharides, and flavonoids. It also supports gene discovery and cloning, thereby laying a foundation for further functional validation and studies on biosynthesis mechanisms.
• This dataset offers SSR markers that may be valuable for breeding programs in *B. chinense* and related species.

## 2 Background

*Bupleurum* species are important medicinal plants. Their roots, known as Bupleuri Radix, have been used in traditional medicine for >2000 years [1]. Among the officially authenticated source species, *B. chinense* DC. is the principal cultivated species in China and the primary commercial source of Bupleuri Radix [2]. Saikosaponins (SS) are the major bioactive constituents of *B. chinense* and are responsible for a wide range of pharmacological activities. In addition, volatile oils, polysaccharides, flavonoids, and other compounds from Bupleuri Radix have been reported to exhibit antimicrobial, antioxidant, hepatoprotective, and other beneficial effects [3]. The biosynthesis of these compounds is regulated by multiple genes involved in the corresponding metabolic pathways. Therefore, obtaining sequence information for functional genes is essential for gene characterization and pathway elucidation.

Previous studies have identified several full-length cDNA sequences encoding *β-amyrin synthase* (*β-AS*), *cytochrome P450 enzymes* (*P450s*), and *UDP-glycosyltransferases* (*UGTs*), which are key genes in the saikosaponin biosynthetic pathway. These sequences were initially obtained from next-generation sequencing (NGS) libraries and subsequently cloned and validated by PCR or RACE [[4], [5], [6]]. However, these approaches have inherent limitations when numerous full-length gene sequences are required. PCR and RACE are labor-intensive, time-consuming, and relatively costly. Although NGS has overcome some of these drawbacks and has been widely applied to generate transcriptomic datasets for *B. chinense* [7], its short-read output, typically <300 bp, makes it difficult to obtain complete transcript sequences directly. Third-generation sequencing technologies, such as single-molecule real-time (SMRT) sequencing by Pacific Biosciences (PacBio), overcomes these limitations by generating long reads, often exceeding 10 kb. Using third-generation sequencing, Li et al. profiled 5- and 15-day-old sprouts of *B. falcatum* L. and identified 130 genes closely involved in saikosaponin biosynthesis [8]. More recently, Wang et al. used the PacBio Sequel platform to obtain full-length cDNAs of ABA-induced bHLH transcription factors from ABA-treated hairy roots of *B. chinense* [9]. Although the genome of *B. chinense*. has been reported, detailed data have not been made publicly available [10]. As a result, a complete and comprehensive set of genes involved in saikosaponin biosynthesis and the biosynthesis of other medicinal compounds has not yet been fully characterized, and full-length sequences for many pathway genes remain unavailable in *B. chinense*.

In this study, we performed full-length transcriptome sequencing of mixed root, stem, and leaf tissues from cultivated *B. chinense* using PacBio SMRT sequencing. The resulting full-length transcriptomic dataset may serve as a valuable reference resource in the absence of a publicly available genome and may facilitate gene function characterization, molecular marker development, and the identification of full-length genes in *B. chinense*.

## 3 Data Description

To characterize the full-length transcriptome profile of *B. chinense*, total RNA was isolated from the mixed root, stem and leaf samples. RNA-seq library was sequenced on the PacBio Sequel platform. The sequencing generated 1.61 GB of circular consensus sequences (CCSs) reads, which had been deposited in the SRA database (PRJNA1494021). Detailed statistics of transcripts and unigenes in the full-length transcriptome of *B. chinense* are shown in Table 1. After clustering and correction, 25,016 high-quality FLNC reads were retained. The longest sequence in each cluster was designated as a unigene, yielding 11,878 unigenes. This dataset provides a valuable resources for obtaining full-length unigene sequences in *B. chinense*.

**Table 1:** Statistics of transcripts and unigenes for the full-length transcriptome of *B. chinense*.

	Transcript subreads	Circular consensus sequence (CCS)	Full-length non-concatemer (FLNC)	Unigene
Total Number	49,403,610	543,207	268,078	11,878
Sequence Base (bp)	78,851,851,671	856,295,942	397,587,759	19,355,819
N50 Length (bp)	1683	1718	1640	1825
Max Length (bp)	362,271	19,555	6964	5735
Average Length (bp)	1596.07	1576.37	1483.1	1629.55
Mean Length (bp)	1380	1512	1421	1570
Min Length (bp)	51	91	51	309

To obtain functional insights into the unigenes, annotation was carried out against six public databases, including the Non-Redundant Protein (NR) database, Gene Ontology (GO), Kyoto Encyclopedia of Genes and Genomes (KEGG), Eukaryotic Orthologous Groups (KOG), Protein families (Pfam) database, and UniProt. In total, 11,448 unigenes were annotated in at least one database (Fig. 1), providing a broad functional overview of the *B. chinense* transcriptome. BLAST searches against the NR database indicated that *Daucus carota* subsp. *sativus* showed the closest sequence similarity to *B. chinense* (Fig. 2), suggesting evolutionary relationships with other species in Apiaceae. Transcription factor analysis identified 690 unigenes distributed across 48 families, among which MYB-related, MYB, AP2, WRKY, bHLH, ERF, and bZIP were the most abundant (Fig. 3). These transcription factors are known to be involved in plant development, stress responses, and secondary metabolism, and may contribute to the regulation of bioactive compound accumulation in *B. chinense*.

**Fig. 1:**
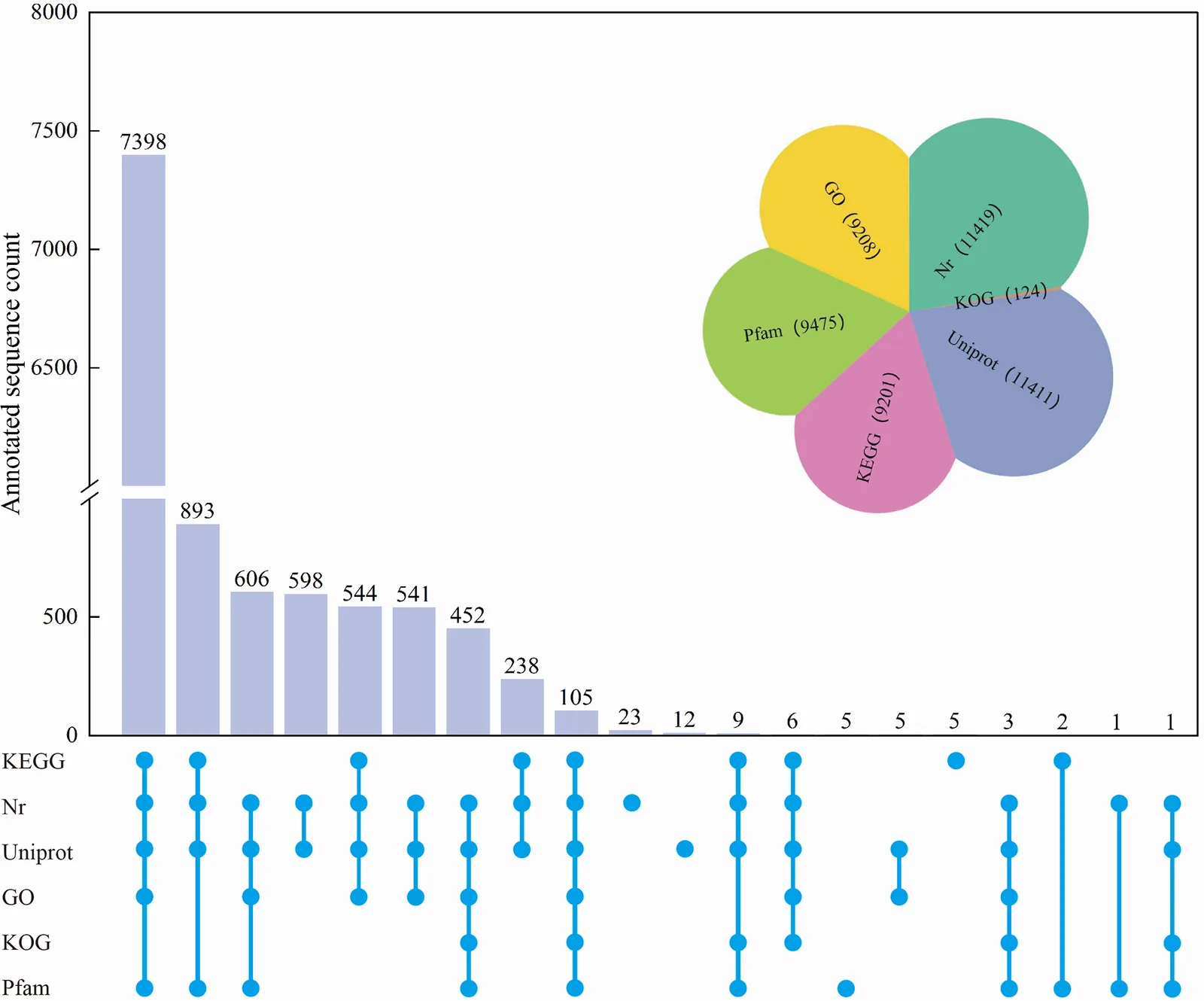
Summary of the functional annotaion of the *B. chinense* transcriptome.

**Fig. 2:**
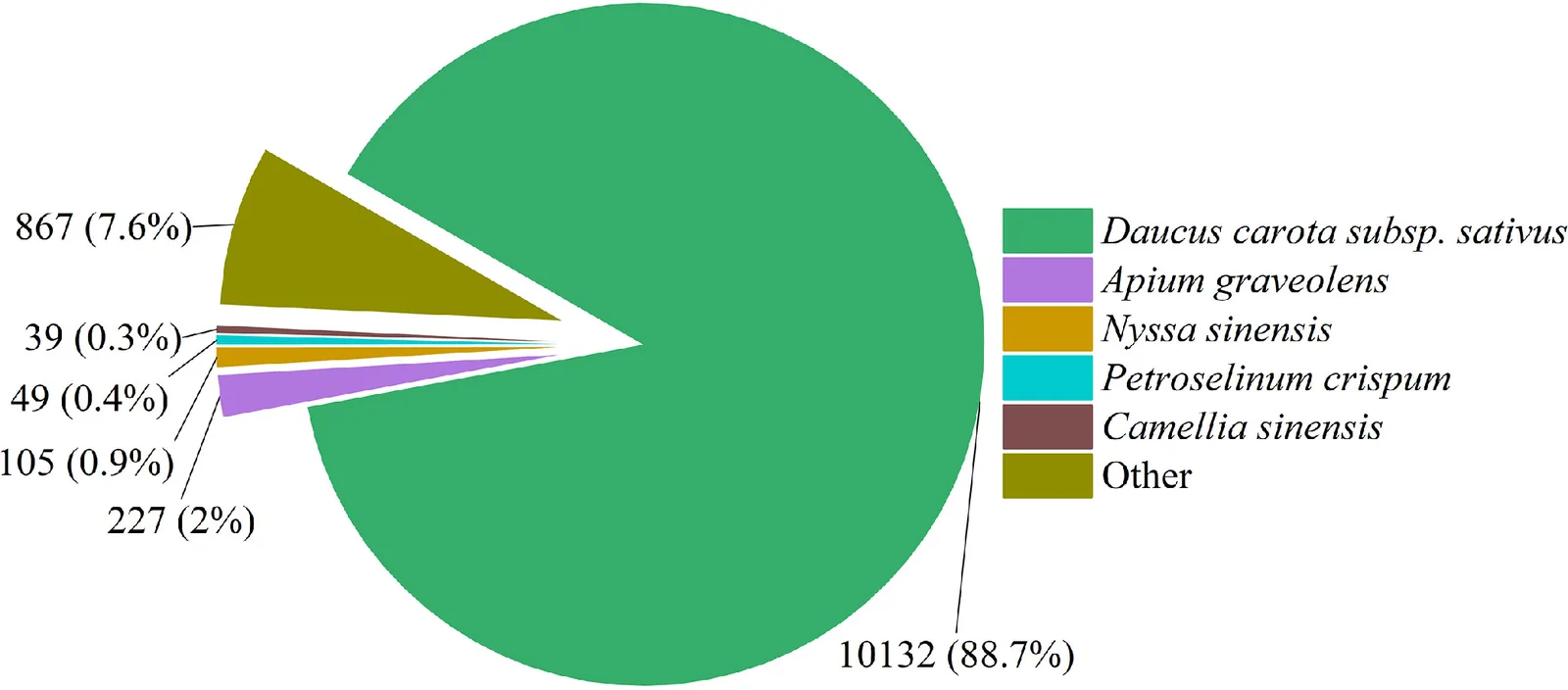
Distribution of homologous species in *B. chinense* with NR annotation.

**Fig. 3:**
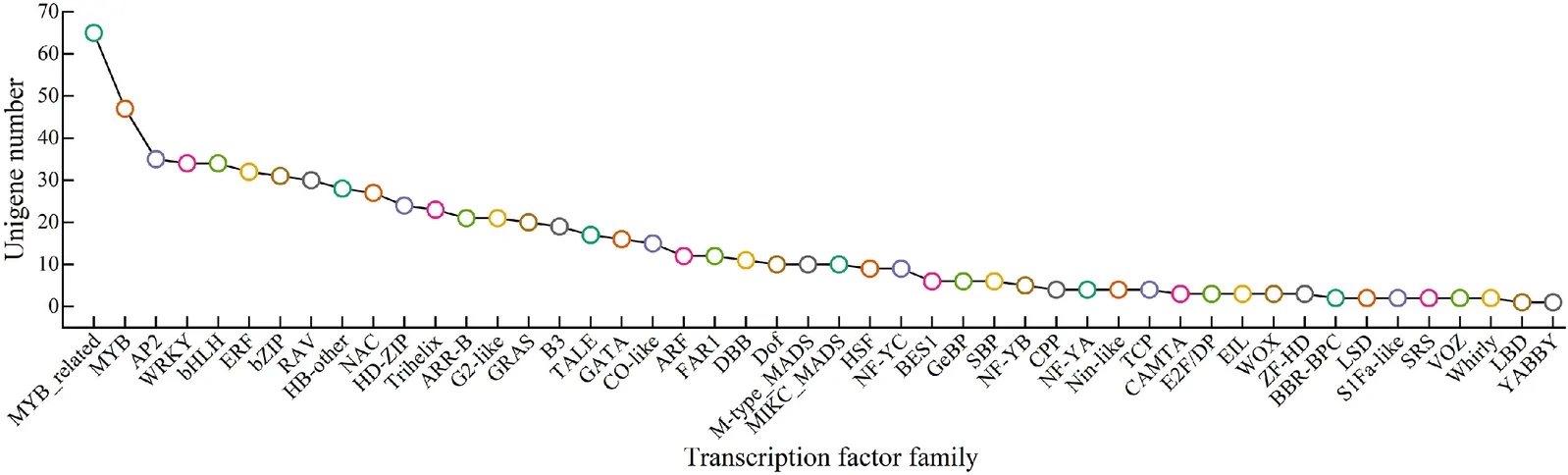
Classification of unigenes encoding transcription factors.

GO annotation showed that the unigenes were mainly associated with fundamental biological processes, membrane-related cellular components, and binding activities (Fig. 4). Unigenes involved in terpenoid biosynthesis were also detected. KEGG analysis further highlighted pathways related to metabolism and secondary metabolite biosynthesis (Fig. 5). In particular, unigenes associated with terpenoid backbone biosynthesis and sesquiterpenoid and triterpenoid biosynthesis were identified, providing valuable candidate genes for elucidating the molecular basis of saikosaponin biosynthesis and for future functional studies, molecular breeding, and metabolic engineering in *Bupleurum* species.

**Fig. 4:**
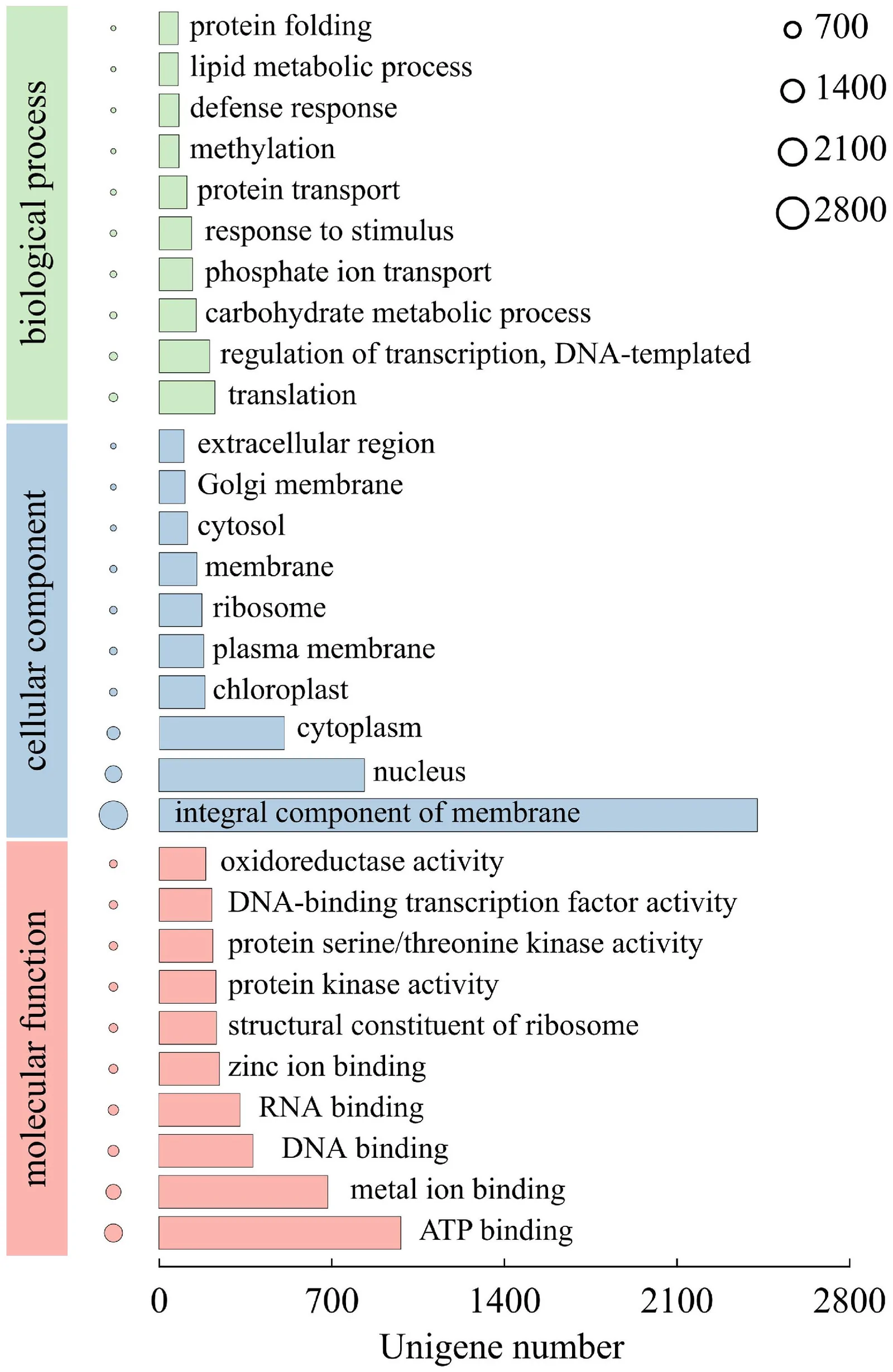
GO category distribution of blast annotated *B. chinense* unigenes.

**Fig. 5:**
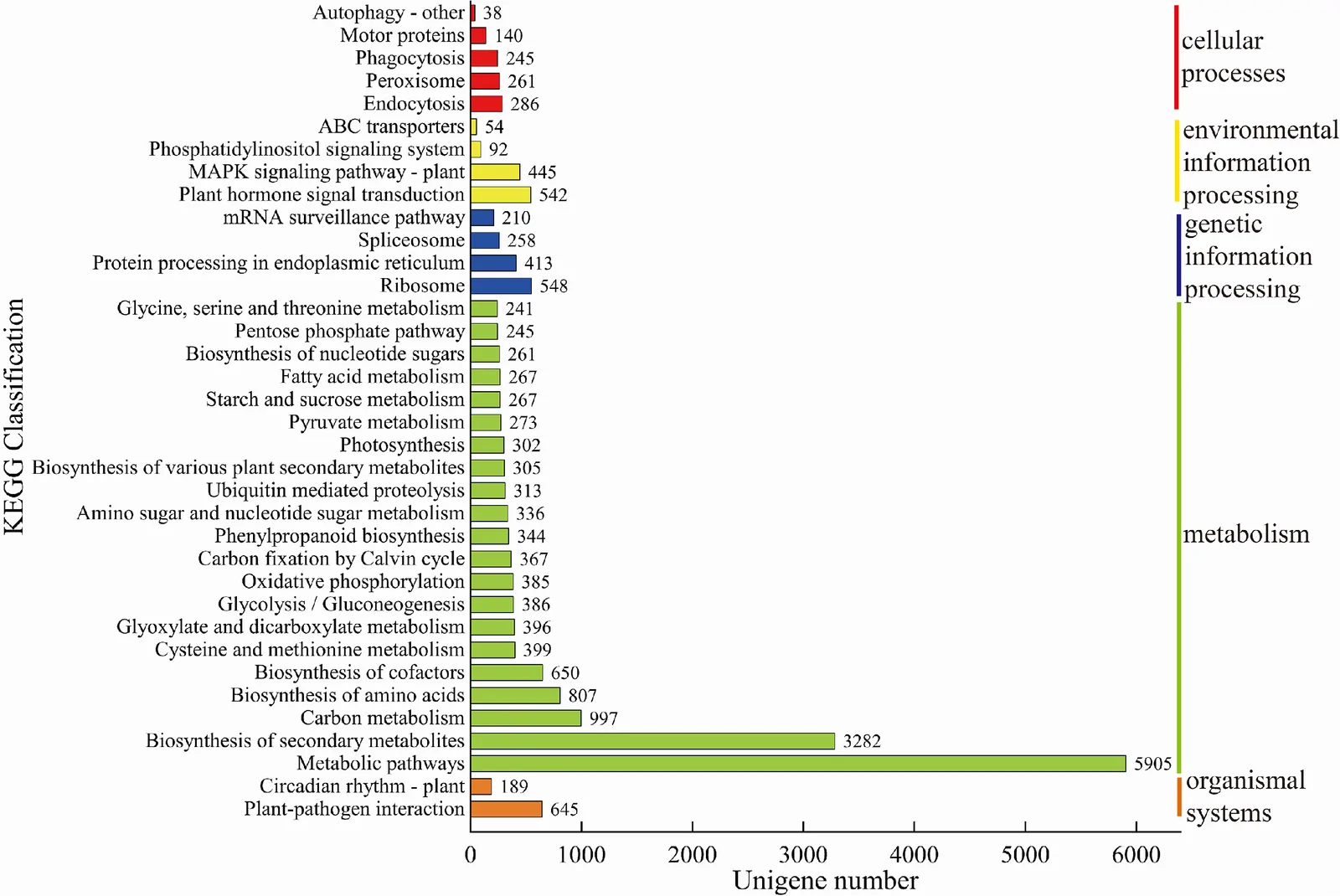
Functional classification of KEGG identified in *B. chinense* transcriptome.

To date, no SSR analysis based on full-length transcriptome has been reported for any species in the genus *Bupleurum*. Using MISA software, we indentified a comprehensive set of SSRs from the full-length transcriptome of *B. chinense*, which were distributed across 4976 unigenes. The SSRs were mainly composed of di-nucleotide repeats, followed by mono- and tri-nucleotide repeats, and compound SSRs were also detected (Table 2). A summary of SSR distribution by repeat number is provided in Table 3. The most frequent motifs was AT/AT (Fig. 6). The SSR markers identified from full-length unigenes provide a valuable molecular resource for genetic diversity analysis, marker-assisted breeding, and germplasm identification in *B. chinense* and related species.

**Table 2:** SSR description.

Item	Number
Total length of Sequence examined (bp)	19,355,819
Total number of SSRs identified	6178
Number of compound microsatellites	900
Total length of SSR	14,660
Relative abundance (SSR/Mb)	377.61
Relative density (bp/Mb)	759.39
Number of SSR containing sequences	4976
Number of sequences containing >1 SSR	1576
Mono-nucleotide	1092
Di-nucleotide	3247
Tri-nucleotide	888
Tetra-nucleotide	33
Penta-nucleotide	4
Hexa-nucleotide	14

**Table 3:** The distribution of SSRs based on the number of repeat units.

Repeat number	Mono	Di	Tri	Tetra	Penta	Hexa	Total	Percentage(%)
5	―	―	529	19	3	9	560	10.61
6	―	758	188	8	1	2	957	18.13
7	―	524	75	2	―	1	602	11.41
8	―	529	30	2	―	―	561	10.63
9	―	422	28	1	―	―	451	8.54
10	440	451	19	―	―	1	911	17.26
11	223	324	4	1	―	―	552	10.46
12	116	122	3	―	―	―	241	4.57
13	83	50	3	―	―	―	136	2.58
14	65	24	2	―	―	―	91	1.72
≥15	165	43	7	―	―	1	216	4.09

**Fig. 6:**
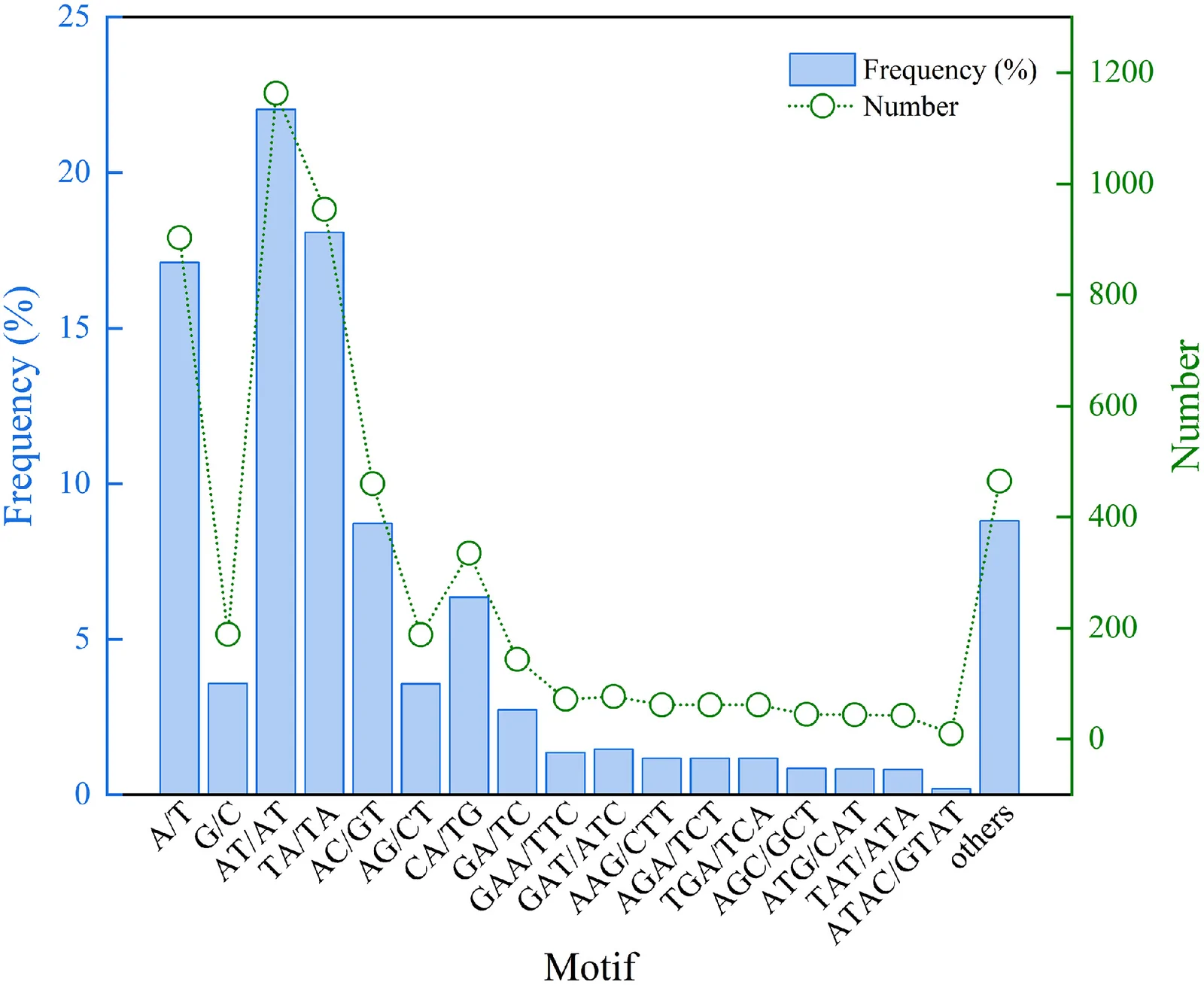
Distribution of SSRs across different repeat motifs in the *B. chinense* transcriptome.

## 4 Experimental Design, Materials and Methods

### 4.1 Plant materials

*B. chinense* cultivar Zhongchai No.2 was used in this study. Seeds were sown on May 1, 2022 in the experimental cultivation field of Chengde Medical University, Chengde, China (41°2′8″ N and 117°56′48″ E, 218 m above sea level), with a row spacing of 40 cm and a plant spacing of 10 cm (Fig. 7). Seedlings were covered with a shading net (four-needle mesh) and the net was removed after the six-leaf stage. Field management of *B. chinense* was conducted in accordance with the group standard of the China Association of Chinense Medicine, namely the “Code of Practice for Good Agricultural Practice of Bupleuri Radix” (T / CACM 1374.128 – 2021).

**Fig. 7:**
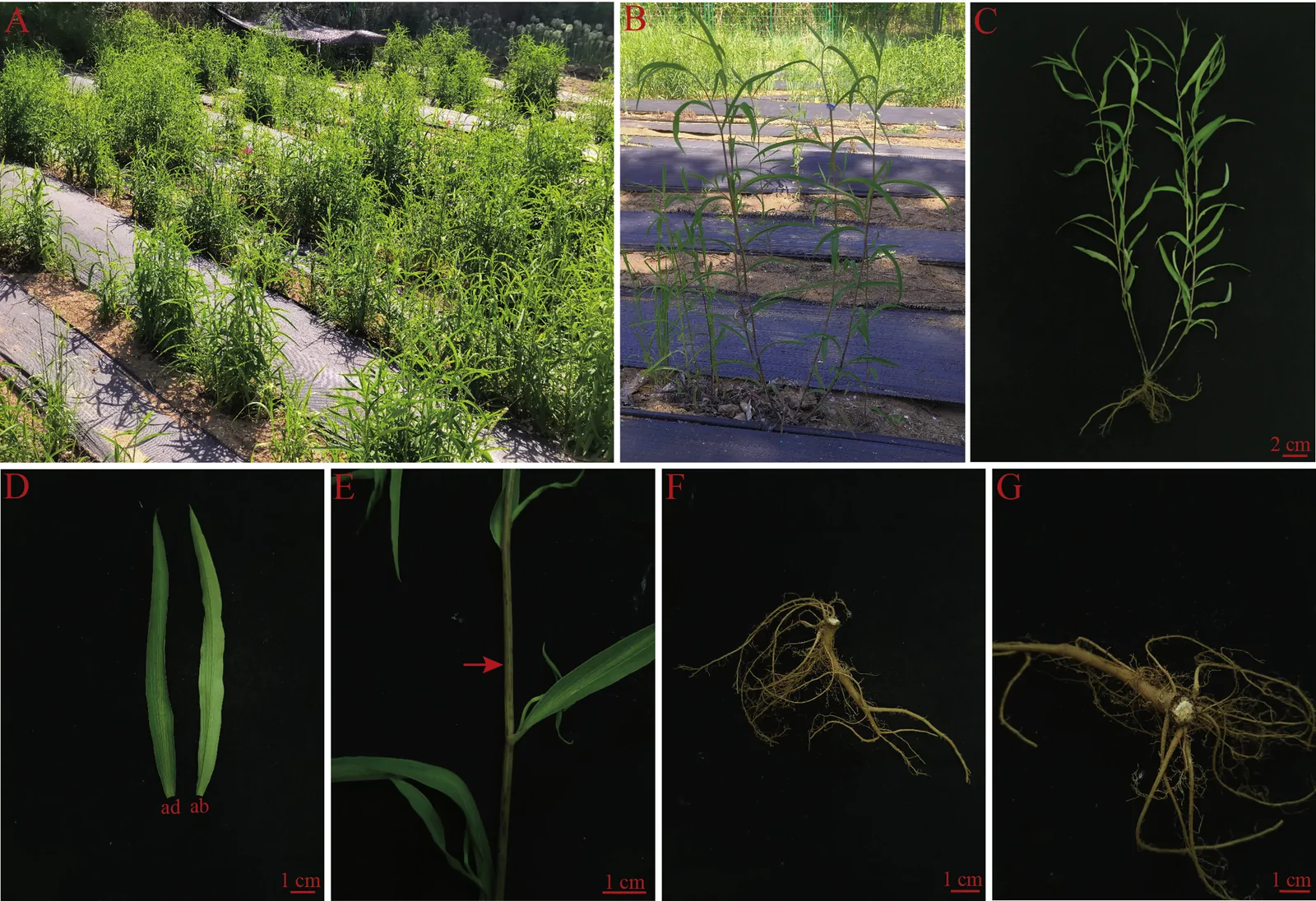
Images of a two-year-old *B. C*hinese. A. Plantation of *B. chinense*. B, C. Whole plant of Zhongchai No. 2. D. Leaf; ad: adaxial surface; ab: abaxial surface. E. Stem. F, G. Root.

Irrigation frequency was determined according to soil moisture conditions and weather conditions, and watering was performed in the evening. During periods of high summer temperatures, irrigation frequency was increased appropriately. In rainy summer periods, drainage was strengthened to prevent root rot caused by the combined stress of high temperature and waterlogging. Weeds were removed regularly. On June 1, 2024, a healthy *B. chinense* plant was randomly selected. Root, stem, and leaf tissues were separated, rapidly frozen in liquid nitrogen, and stored at − 80 °C until further use.

### 4.2 Total RNA extraction, library preparation, and sequencing

Total RNA was extracted from root, stem and leaf tissues using an E.Z.N.A.® HP Total RNA Kit (Omega Bio-tek, USA) according to the manufacturer's instructions. Only RNA samples with RIN ≥ 7.0 were retained. RNA purity and concentration were determined by a Nanodrop 2000 spectrophotometer (Thermo Fisher Scientific, USA) and a Qubit 3 Fluorometer (Thermo Fisher Scientific, USA), respectively.

Equal amounts of high-quality total RNA from the three tissues were pooled prior to cDNA library preparation to maximize transcript diversity and increase the likelihood of capturing low-abundance full-length isoforms. This strategy was chosen because the objective of the study was to generate a comprehensive reference transcriptome rather than tissue-specific expression profiles.

Full-length cDNA was synthesized using the Clontech SMARTer™ PCR cDNA Synthesis Kit (Clontech Laboratories, Palo Alto, CA, USA) following the manufacturer’s instructions. Briefly, the oligo(dT) primer annealed to the poly(A) tail of eukaryotic mRNA during first-strand synthesis, and the SMARTer template-switching mechanism enabled the addition of defined sequences to both ends of the cDNA, thereby enriching for full-length transcripts. The resulting full-length cDNA was amplified by PCR, purified with PB magnetic beads, and quantified using a Qubit 3.0 fluorometer. To improve library quality, cDNA fragments smaller than 1 kb were removed by size selection with PB bead. The size-selected cDNA was then subjected to end repair and ligated to SMRTbell hairpin adapters to generate SMRTbell templates. Unligated DNA molecules and other non-circularized products were removed by exonuclease digestion, followed by an additional PB bead purification step. Library quality was assessed by Qubit 3.0 quantification and fragment size analysis using an Agilent 2100 Bioanalyzer. Only libraries meeting the required quality criteria were used for sequencing. SMRT sequencing was performed on a PacBio Sequel II platform. The library was sequenced using 1 SMRT cells, with a Sequel Ⅱ Binding Kit 2.0 chemistry.

### 4.3 Transcriptome assembly

Raw PacBio sequencing data were processed using SMRT Link (v11.0) and the Iso-Seq3 pipeline. Polymerase reads were split into subreads, which were used to generate circular consensus sequences (CCSs). CCS reads were screened for the presence of 5′ and 3′ primers and poly(A) tails. Reads containing both primers and lacking concatemeric structures were classified as full-length non-concatemeric (FLNC) reads. FLNC reads were clustered and polished using the Iso-Seq3 cluster module. Redundant sequences were removed using CD-HIT (v4.8.1) with a 99% similarity threshold [11], and the resulting high-quality non-redundant full-length transcripts were retained for downstream analyses.

### 4.4 Functional annotation

To annotate the structural and functional features of the newly identified transcripts, coding sequences (CDSs) were predicted using TransDecoder (v5.5.0) with the parameters -m 50 and –single_best_only (http://transdecoder.github.io). Functional annotation was then performed against the NR (downloaded on 2024–02–07), Pfam (release 30.0), UniProt (release 2021_01), KEGG (release 97.0), GO (release 2021–01–01), and KOG (last updated in 2003) databases using both sequence similarity- and domain-based approaches. Predicted protein sequences were aligned against the NR and UniProt databases using DIAMOND blastp (v2.0.6.144) with an E-value cutoff of 1e-5 [12]. Conserved domains were identified using hmmscan (v3.3.2) against Pfam with an E-value threshold of 0.01 [13]. KEGG Orthologs were assigned using kofam_scan (v1.3.0) with parameter -E 1e-5. GO annotations were summarized into biological process, cellular component, and molecular function categories. Species distribution of NR hits was also analyzed.

### 4.5 Analysis of transcription factors and simple sequence repeats (SSRs)

Transcription factors were identified in B. *chinense* unigenes using PlantTFDB (v5.0) [14]. MISA (v.1.01) [15] was used to retrieve the SSR loci from the full-length transcriptome of *B. chinense*. The search criteria were as follows: the minimum repeat numbers for mono-, di-, tri-, tetra-, penta-, and hexanucleotide SSRs were 10, 6, 5, 5, 5 and 5, respectively.

## Limitations

None.

## Ethics Statement

The authors have read and followed the ethical requirements for publication in Data in Brief and confirmed that the current work does not involve human subjects, animal experiments, or any data collected from social media platforms.

## CRediT Author Statement

**Yanxia Gao:** Methodology, Investigation, Writing - Original draft preparation. **Binbin Wang:** Data curation, Software. **Jing Wang:** Visualization. **Yan Xie:** Methodology, Visualization. **Jiahui Wang:** Visualization. **Lan Yang:** Visualization. **Gaixia Zhang:** Data curation, Investigation. **Hui Wang:** Conceptualization, Supervision, Data curation, Writing-Reviewing and Editing.

## Data Availability

National Center for Biotechnology InformationFull-length transcriptome sequencing of Bupleurum chinense DC. (Original data)
